# Attitudes of healthcare workers at a District in KwaZulu-Natal regarding institutionalisation of antimicrobial stewardship

**DOI:** 10.4102/sajid.v40i1.677

**Published:** 2025-01-17

**Authors:** Andile P. Dlungele, Lehlohonolo Mathibe

**Affiliations:** 1 Division of Pharmacology (Therapeutics), Discipline of Pharmaceutical Sciences, School of Health Sciences, University of KwaZulu-Natal, Durban, South Africa

**Keywords:** antimicrobial stewardship, antimicrobials, antimicrobial resistance, knowledge, attitudes

## Abstract

**Background:**

The Antimicrobial Stewardship (AMS) programme is one of the strategic initiatives of the World Health Organization’s global action plan to reduce antimicrobial resistance (AMR). In sub-Saharan countries, there is insufficient scientific evidence regarding healthcare providers’ perception of institutionalisation of ASPs as a strategy to reduce AMR.

**Objectives:**

This study investigated the knowledge, attitudes and practices of healthcare workers in the uMgungundlovu District regarding the enablers and barriers to institutionalisation of AMS programmes in public health settings.

**Method:**

This was a prospective, cross-sectional and a 5-point-Likert-scale (1 = minimal; 2 = limited; 3 = average; 4 = good and 5 = comprehensive) questionnaire-based arm of a larger mixed-methods study. Voluntary participants were purposively recruited from hospitals and community health centres in the uMgungundlovu District, South Africa.

**Results:**

Forty-nine (*N* = 49) participants were recruited. That is, 33% (*n* = 16), 28% (*n* = 14), 25% (*n* = 12) and 14% (*n* = 7) were nurses, pharmacists as well as pharmacist interns, medical practitioners and healthcare managers, respectively. Eighty percent (*n* = 40; median score 5, interquartile range [IQR] 4–5) and 67% (*n* = 33; median score 4, IQR 3–4) of participants felt that they had a key role in reducing antibiotic resistance; and good knowledge of antimicrobials as well as AMS programmes, respectively. However, participants who had been employed for less than 12 months across all the facilities had ‘limited’ knowledge (median score of 2; IQR 1–3.5) of antimicrobials and AMS programmes.

**Conclusion:**

Healthcare workers in public healthcare settings in the uMgungundlovu District knew how crucial their roles were in reducing AMR.

**Contribution:**

This study highlights the need for employment experience and adequate healthcare service providers as critical factors for the successful institutionalisation of AMS programmes in public health facilities.

## Introduction

Antimicrobial resistance (AMR) is a global public health concern requiring immediate response.^1^ In 2019, there were estimated 4.95 million and 27.3 per 100 000 deaths associated with AMR around the world and in sub-Saharan Africa, respectively.^2^ Therefore, to combat this threat, the World Health Organization (WHO) adopted the Antimicrobial Stewardship (AMS) programme as one of the strategic initiatives to reduce AMR globally.^3^ The AMS programme are intentional and specific interventions which advance rational antimicrobial use as a means to achieve the best patient outcomes.^1,4,5^

Healthcare workers are well-positioned at the hospital and community’s interface to play meaningful roles in the local AMS programmes.^6^ However, a global survey on antimicrobial stewardship activities in Africa revealed that only 14% of respondents had any form of AMS programme in place.^5,7^ Joseph O Faredare and colleagues reported that there were insufficient scientific data regarding the existence and functional of AMS programmes in Nigerian hospitals.^8^ Furthermore, according to Essack and colleagues, only 4.3% of countries in the WHO Africa region have national AMR plans, while 14.9% have national infection prevention and control policies.^9^ The positive impact of a functional AMS programmes has been demonstrated in South Africa.^4^ However, optimal strategies for AMS programmes, including membership and activities, have yet to be fully investigated.^8^ The main barriers to the implementation of AMS programmes in almost all public and private hospitals have been inadequate infectious diseases expertise and resources; additionally, in large hospital networks, the geographical distribution of these institutions has also hindered implementation.^5^ In South Africa, anecdotal evidence also suggests that the non-existence as well as indifference of the authorities regarding the institutionalisation of AMS programmes are some of the major obstacles preventing the effective implementation of WHO’s noble strategic objective. Therefore, this study investigated the knowledge and attitudes of healthcare workers as well as barriers which hindered the institutionalisation of AMS programmes in public healthcare settings in the uMgungundlovu District, KwaZulu-Natal (KZN) province, South Africa.

## Research methods and design

### Study design

This was a prospective, cross-sectional and 5-point-Likert-scale (1 = minimal; 2 = limited; 3 = average [not sure or not applicable]; 4 = good and 5 = comprehensive) questionnaire-based arm of a larger mixed-methods study. Voluntary participants were purposefully recruited from the uMgungundlovu District, KZN province, South Africa. The construct of the questionnaire was informed by the items in the WHO practical tool kit.^10^ Briefly, the questionnaire had three sections: (1) the demographic profile of participants, (2) structured close-ended questions on participants’ perceptions regarding the use of antimicrobials as well as their role in combating AMR and (3) participants’ knowledge of antibiotics and the causes of AMR. Microsoft Excel was utilised to capture quantitative data and to calculate the proportions. Graph Pad Prism version 5 was used to calculate medians and their interquartile ranges (IQRs).

### Study settings and ethics approval

Data collection took place at three district hospitals, one regional hospital, one tertiary or teaching hospital, four specialised hospitals and three CHCs. Briefly, the district hospitals receive referrals from the three CHCs and 49 primary healthcare clinics. Patients who require management at a higher level of care are referred from district hospitals to a regional hospital. The specialised hospitals provided management for specialities such as multi-drug-resistant tuberculosis (MDR-TB) and psychiatry.^11^ The uMgungundlovu District provides healthcare services to about 10% (*n* = 1 281 848) of the population in the KZN province.^12^

### Ethical considerations

Ethical clearance to conduct this study was obtained from the University of KwaZulu-Natal Ethics Committee and the Department of Health KZN province (reference no.: BREC/00003100/2021).

## Results

### Demographic characteristics of participants

Forty-nine (*N* = 49) participants were recruited. That is, 33% (*n* = 16), 28% (*n* = 14), 25% (*n* = 12), and 14% (*n* = 7) were nurses, pharmacists/pharmacist interns, medical practitioners and managers, respectively. During the period of this study, 18% (*n* = 9), 41% (*n* = 20), 27% (*n* = 13) and 14% (*n* = 7) of participants were employed for < 12 months, 12 months to 5 years, > 5 years to 10 years and > 10 years, respectively.

### Participants’ perceptions regarding their knowledge of antimicrobials and Antimicrobial Stewardship Programmes

As depicted in Table 1, 67% (*n* = 33) of participants felt that they had ‘good’ knowledge (median score of 4; IQR 3–4) of antimicrobials as well as AMS programmes. Eighty per cent (*n* = 40) of participants agreed or strongly agreed that they have a key role to play in reducing AMR (median score of 5; IQR 4–5). However, participants who had been employed for less than 12 months, across all the facilities, felt that they had ‘limited’ knowledge (median score of 2; IQR 1–3.5) of antimicrobials as well as AMS programmes. There were medians of 4 (IQR 3–4) for participants who had been employed for more than 12 months, and there were no differences across the various professions. Departmental meetings were the main source of information on AMS programmes for 80% (*n* = 39) of participants – this was followed by emails (25%; *n* = 12) and workshops (20%; *n* = 10).

**TABLE 1:** Participants’ perceptions regarding the use of antimicrobials and their roles in combating of antimicrobial resistance.

Perception	Median	IQR	Disagree or strongly disagree		Not sure		Agree or strongly agree	
			%	*n*	%	*n*	%	*n*
Every person treated with antibiotics is at an increased risk of antibiotic-resistant infection	4	2–4	27	13	6	3	67	33
I have a key role to play in controlling antibiotic resistance	5	4–5	6	3	6	3	80	40
The personality and behaviour of patients significantly influence my dispensing practices	2	2–4	57	29	10	5	24	12
Patients may feel better if you prescribe antibiotics to satisfy their demands and expectations	2	1–3	69	35	10	5	12	6

IQR, interquartile range.

As shown in Table 2, 88% (*n* = 43) of participants strongly disagreed that antibiotics are effective against viruses, and 96% (*n* = 48) strongly agreed that unnecessary use of antibiotics makes them ineffective. All participants (100%, *n* = 49) knew that overuse of antibiotics promotes AMR, and 76% (*n* = 38) were aware that there is a connection between irrational prescribing, dispensing or administering of antibiotics and the emergence and spread of antibiotic-resistant bacteria.

**TABLE 2:** Participants’ knowledge of antibiotics and the causes of antimicrobial resistance.

Knowledge	Median	IQR	Disagree or strongly disagree		Not sure		Agree or strongly agree	
			%	*n*	%	*n*	%	*n*
Antibiotics are effective against viruses	1	1–2	88	43	2	1	10	5
Antibiotics are effective against common colds and flu	1	1–2	78	38	2	1	18	9
Unnecessary use of antibiotics makes them ineffective	5	4–5	4	2	0	0	96	48
Antibiotic resistant bacteria can spread from person to person	4	4–5	18	9	4	2	76	37
Every person treated with antibiotics is at an increased risk of antibiotic-resistant infection	4	2–4	27	13	6	3	67	33
Widespread overuse of antibiotics promotes antimicrobial resistance	5	4–5	0	0	0	0	100	49
The usage of broad-spectrum antibiotics promotes antibiotic resistance	4	4–5	4	2	4	2	90	45
Poor infection control in hospitals spreads antimicrobial resistance	4	4–5	16	8	4	2	80	40
Patient poor adherence causes antibiotic resistance	5	4–5	0	0	2	1	98	48
Poor antibiotic handling methods promote antibiotic resistance	4	3–5	16	8	12	6	71	36
Antibiotics could be used to prevent bacterial infection in patients with viral infection	2	1–4	61	30	0	0	22	11
There is a connection between my prescribing, dispensing or administering of antibiotics and the emergence and spread of antibiotic-resistant bacteria	4	4–5	10	5	4	2	76	38
It is better to stop antibiotics as soon as a patient feels better	2	1–4	57	27	8	4	22	11

IQR, interquartile range.

The main barriers which were perceived as hindrances to the effective institutionalisation of AMS programmes were insufficient medical practitioners (59%, *n* = 29), staff shortage (57%, *n* = 28), AMS programmes not being made a priority (41%, *n* = 20), and insufficient funds (12%, *n* = 6).

## Discussion

This is the first study to report on the knowledge, attitudes and barriers encountered by the healthcare workers in their endeavours to institutionalise AMS programmes in the uMgungundlovu Health District, the second-densely populated region in KZN province, South Africa.^13^ The findings of this research suggest that healthcare workers have good knowledge of antimicrobials and their role in the institutionalisation of AMS programmes. In a similar study, and in support of the findings of this study, Catanzaro recently reported that over 80% of staff nurses in Georgia, Louisiana as well as Pennsylvania in the United States had positive attitudes towards their role as stewards of antimicrobial therapy.^2^ This was similar to a study which was conducted at a referral hospital in Malawi.^14^ Therefore, healthcare workers are keen on the institutionalisation of AMS programmes despite numerous barriers which continue to be a hindrance. The findings of our study suggest that insufficient medical practitioners in public healthcare settings was the main barrier which prevented efficient institutionalisation of AMS programmes. Similarly, an AMS situational analysis study across all public sector hospitals in KZN conducted by Chetty and colleagues reported a lack of human resources, low clinician buy-in and financial restrictions on antimicrobial research and education as other barriers.^15^

## Conclusion

Healthcare workers in public healthcare settings in the uMgungundlovu District were aware of the role they can play in reducing AMR. However, there are institutional barriers that systematically continue to hinder the effective implementation of AMS programmes in healthcare settings such as the uMgungundlovu District. Therefore, regular in-service educational programmes, especially for newly appointed professionals, as well as investment in human resources in public healthcare settings will lead to the effective institutionalisation of AMS programmes and sustained triumph against AMR.
